# Gender Differences in Emotional Responses to Cooperative and Competitive Game Play

**DOI:** 10.1371/journal.pone.0100318

**Published:** 2014-07-01

**Authors:** J. Matias Kivikangas, Jari Kätsyri, Simo Järvelä, Niklas Ravaja

**Affiliations:** 1 Department of Information and Service Economy, Aalto University School of Business, Helsinki, Finland; 2 Department of Media Technology, Aalto University School of Science, Espoo, Finland; 3 Department of Social Research and Helsinki Institute for Information Technology, University of Helsinki, Helsinki, Finland; University of Tuebingen Medical School, Germany

## Abstract

Previous research indicates that males prefer competition over cooperation, and it is sometimes suggested that females show the opposite behavioral preference. In the present article, we investigate the emotions behind the preferences: Do males exhibit more positive emotions during competitive than cooperative activities, and do females show the opposite pattern? We conducted two experiments where we assessed the emotional responses of same-gender dyads (in total 130 participants, 50 female) during intrinsically motivating competitive and cooperative digital game play using facial electromyography (EMG), skin conductance, heart rate measures, and self-reported emotional experiences. We found higher positive emotional responses (as indexed by both physiological measures and self-reports) during competitive than cooperative play for males, but no differences for females. In addition, we found no differences in negative emotions, and heart rate, skin conductance, and self-reports yielded contradictory evidence for arousal. These results support the hypothesis that males not only prefer competitive over cooperative play, but they also exhibit more positive emotional responses during them. In contrast, the results suggest that the emotional experiences of females do not differ between cooperation and competition, which implies that less competitiveness does not mean more cooperativeness. Our results pertain to intrinsically motivated game play, but might be relevant also for other kinds of activities.

## Introduction

Gender differences in competitiveness have been widely studied since after Darwin (see [1]), and males' greater tendency to compete has been reported in various contexts, such as career choices and labor market [2], [3], negotiations and bargaining [4], [5], economic experiments [6], [7], and sports [8]. By contrast, females have been considered to be more cooperative (e.g., [9]–[12]). However, despite the difference in the behavioral preference, whether there is also a difference between genders in emotions behind the behavior has not been previously investigated.

When looking at the question regardless of gender, there is reason to expect that competition should be experienced more negatively than cooperation. According to appraisal theory, negative emotions are elicited by situations that are inconsistent with one's own goals [13]—and given that competitive situations include opposing goals between the participants, competition is by definition a threat of one's goals being blocked. Furthermore, evolutionary psychology suggests that in this regard, humans have evolved positive emotions to facilitate survival through cooperation, and negative emotions to defend against those who do not cooperate (e.g., [14]) and to help in competition for resources [15]. Consequently, competition should elicit more negative emotions than cooperation. Although there is no direct empirical evidence for differential emotional responses to competition and cooperation, for example students have been found to experience teaching more negatively when it is organized with competitive rather than cooperative goals [16]. Neuroscientific studies have indicated that in economic decision making games, cooperation is associated with reward system activations [17] and noncooperation is associated with neural activations suggestive of negative emotions [18].

Although theoretical considerations would suggest that competition is associated with negative emotions, during activities such as sports and games people in fact seem to derive great enjoyment from competition and actively seek it [19]–[21](cf. [22]). An explanation for this apparent discrepancy might be found in the motivational source for competition; that is, whether it is chosen voluntarily, constituting intrinsic motivation, or it is forced by external factors (extrinsic motivation). Self-determination theory [23] posits that voluntary participation in a challenging activity increases feelings of competence and autonomy as the goals are set by the person himself or herself, resulting in a high level of enjoyment. Although the motivation literature largely connects competition to extrinsic motivation and less positive emotions, competition can be subjectively viewed as intrinsically motivated as well [23], [24]. In the terms of appraisal theory, if the person is motivated not by achieving victory and some extrinsic goals attached to it, but by playing itself, voluntary participation in competitive game play reinforces rather than blocks the player's goals.

Returning to gender differences, research evidence as described above suggests that males show a behavioral preference for competition over cooperation, whereas females prefer cooperation over competition—in fact, the (limited) evidence seems to suggest that these preferences hold regardless of whether the activity is extrinsically or intrinsically motivated (see [6], [8], [10], [25]–[28]). Because preferences are based on emotions [29], this leads to the question whether males and females also show different emotional responses to competitive and cooperative activity modes; that is, whether males show more positive emotional responses during competition than females, and whether females show more positive responses during cooperation than males.

### Present studies

We conducted two studies where we compared the emotional responses elicited by cooperative (participants have congruent goals – both win or lose together) and competitive (participants have mutually exclusive goals – if one wins, the other loses) game play. We used digital games for the research task because they provide an ecologically valid and culturally widespread intrinsically motivated activity in the modern Western world, and they also have the benefit of being relatively easily adaptable for experimental purposes—unlike, for example, sports, which have more physical and practical limitations. One previous study focusing on emotions elicited by competitive and cooperative games has already suggested that competition is more enjoyable (more positive emotion) than cooperation [30]; however, this study did not consider differences between males and females. As digital game playing is more popular among males than females [31] and females tend to dislike certain features of games (i.e., violence, negative gender stereotypes, and lack of social interaction; see [32]), we paid special attention to selecting games which would not be disproportionately more preferred by one gender.

In addition to self-report questionnaires, we used psychophysiological methods to study emotional experiences. There is compelling evidence that facial electromyography (EMG), electrodermal activity, and cardiac indices can be used to assess emotional valence, arousal, and attention, respectively [33], [34]. Within the last decade these measurements have also been successfully applied to study emotions during digital game play ([35]–[38]; see also [39], [40]). To summarize, facial EMG activity over zygomaticus major and orbicularis oculi (ZM and OO, activated when smiling) muscle areas are generally considered good indices for positive emotions, and activity over corrugator supercilii (CS) muscle area (activated when frowning) an index for negative emotions, while electrodermal activity (EDA) is a widely used measure for assessing arousal originating from physiological (i.e., sympathetic nervous system) activations [33], [34]. In addition, heart rate (HR) is often used to assess arousal, although the dual innervation of heart by both the sympathetic and parasympathetic branches of the autonomous system makes the interpretation of HR changes difficult, particularly when using a more complex stimulus (see [39]).

### Hypotheses

Based on the above considerations, we formulated the following hypotheses:

*H1: Males will show higher positive emotion (as assessed by ZM and OO EMG activity and self-reports) in competitive than in cooperative game play.*

*H2: Females will show higher positive emotion (as assessed by ZM and OO EMG activity and self-reports) in cooperative than in competitive game play.*

Given that there is evidence for both greater emotional expressiveness [41], [42] and more intense emotional experiences [43] in females than males, greater overall facial EMG activation could be expected for females. However, this should not affect the differences between the competitive and cooperative modes.

Recent evidence indicates a degree of independence between positive and negative emotions (see [44]). Therefore it is possible that, as suggested by appraisal theory (negative emotions elicited by one's goals being blocked in competition) and some empirical findings [16], there would be more negative responses to competition (compared to cooperation) regardless of possible simultaneous positive responses. On the other hand, digital games are likely to enable intrinsic motivation when they are played by participants who voluntarily play games in their free time [45]. Consequently, given that the primary goal of intrinsically motivated participants is simply to enjoy playing the game rather than to win against the opponent, they might show no negative responses to competition. Considering these conflicting predictions, but seeing that there is no strong empirical evidence for either one, we present a research question:

*RQ1: Is there a difference in negative emotion (as assessed by CS EMG activity and self-reports) between competition and cooperation, and is it associated with gender?*

Finally, previous studies have reported that competition elicits higher self-reported arousal [30] and cardiovascular activity [46] than cooperation. Although males have been reported to be more responsive to changes in arousal [41], neither of these previous studies have given evidence that would support differences between the genders during competition and cooperation. This leads us to the following hypothesis:

*H3: Both genders will show higher arousal (as assessed by EDA, HR and self-reports) in competitive than in cooperative game play.*

## Experiment 1

The first experiment was conducted using two activity modes, competitive and cooperative, during a digital game. In addition, we sought to demonstrate that our digital game playing results were not confounded by the unfamiliarity of the research laboratory environment (see [47]), as the ecological validity of the research environment may be of particular importance (cf. [25]). In order to address this issue, we repeated the experimental procedure both in laboratory and in a more familiar environment (participant's home).

### Methods

#### Participants and ethics statement

Participants were 48 (18 female) volunteers, ranging from 18 to 34 years of age (M = 24.0, SD = 4.09), who played digital games on a regular basis (at least 4 hours per month). The number of female participants was smaller due to the difficulty of finding female volunteers with sufficient familiarity with digital games; however, we considered the ratio of females versus males to lie within acceptable limits. The participants were recruited in same-gender dyads by advertisements in gaming-related websites, student mailing lists, and student organizations. The dyads volunteered together, so they knew each other before the experiment. Although males and females were both considered regular players, when the previous experience of the particular game used in this experiment (Bomberman) was surveyed, males had more experience with it than females. This difference was taken into account in our statistical analyses.

We have complied with the APA's ethical standards and the Declaration of Helsinki in conducting the experiment. Participants were explicitly reminded that they could withdraw from the study at any time without negative consequences. Because the study did not concern medical research, the need for formal approval was, in accordance with Finnish law, waived by the ethics review board of our university. All participants received three movie tickets as compensation for their participation.

#### Materials

The game used was Bomberman (Hudson Entertainment, Redwood Shores, CA, 2006), played on PlayStation Portable handheld game console (Sony Computer Entertainment, Tokyo, Japan). Bomberman is a classic action game, in which two or more players are situated in a small maze and each player is using bombs to clear new routes and to attempt to eliminate the other players. The game was played in two two-player teams, with the two human participants assigned either to the same team (in cooperation) or different teams (in competition), while the remaining two characters were controlled by the computer. With the exception of team arrangements, the game was identical in both modes. Eliminating the opponent team was the only explicit goal in the game, and the success in this task (number of matches won) was displayed between the matches. For one match in the game, the last player in the maze was declared the winner (in the rare case of two players blasting each other at the same moment, a tie was declared). In our games, each match lasted between two and five minutes. Bomberman employs cartoonish graphics from isometric view and happy music and sounds. It does not contain realistic depictions of violence or negative gender stereotypes, and the nature of multiplayer game makes it inherently social.

#### Design and procedure

The experiment had a 2×2×2 mixed design, with gender (male, female) as the between-subjects factor, and activity mode (competition, cooperation) and environment (laboratory, home) as the two within-subjects factors. The experiment took place in two different locations: laboratory and the home of one of the two participants. In both locations, a practice session was followed by a rest period of 5 min (during which baseline physiological measurements were performed), and two 8-min play periods, one in competitive and one in cooperative mode. The participants played as many matches they could during the 8-min playing periods.

The order of environments was randomized, and the order of modes was counterbalanced across them (chosen randomly from two orders: BA AB or AB BA). When playing at a participant's home, the environment was ensured to be free of distractions (e.g., other people), and the illumination and immediate surroundings (such as chairs) were adjusted for comfortable play. In both locations the participants were seated next to each other, while the experimenter was located in an adjacent room during the actual measurements. The duration of the whole experiment was three to four hours, including the short trip between the locations, all conducted within a single day.

#### Physiological data acquisition

The physiological signals and environmental data were recorded from both players during all the play periods with two Varioport-B portable recorder systems (Becker Meditec, Karlsruhe, Germany), and preprocessed using Matlab software (version 2011a) and ANSlab (version 2.4, University of Basel, Germany) toolbox. All wirings were taped in place and attached to the recording device placed on a belt, not too tightly so that the participants' movements were not hindered. The electrodes were attached before the first practice session in the first location, and the recordings were run over the whole experiment, while baseline and play periods were marked with a hardware marker button. Baseline activity levels were recorded for all physiological signals, so that individual baseline differences could be partialled out in our analyses.

Facial EMG activity was recorded from the left CS (brow), ZM (cheek), and OO (near the corner of the eye) muscle regions. The electrodes were attached so that they would not enter the field of vision. Electrode wires were taped to the skin behind the ear and down to the back of the neck, so that they would not hinder any head movements. We used surface Ag/AgCl electrodes with a contact area of 4 mm diameter. Facial EMG signal was sampled at 1024 Hz at 57–390 Hz frequency range, rectified, and smoothed with a linear phase FIR filter using the Kaiser window method (101 coefficients, low-pass cutoff frequency 40 Hz). EDA signal was recorded using a constant 0.5 V voltage across Ag/AgCl electrodes with a contact area of 4 mm diameter, and sampled at 32 Hz. Electrodes were attached to the medial phalanges of the ring and little fingers of the participant's left hand using self-adhesive electrode collars and electrolyte gel. Ring and little fingers were used, instead of the more typically used index and middle fingers, because holding the game console left ring and little fingers free. Skin conductance level (SCL, an average over the whole period) was derived from the EDA signal. ECG was recorded with three electrodes in a modified Lead 2 placement, sampling at 512 Hz. Heart rates were extracted by identifying R-peaks from the signal in ANSlab software package (http://www.anslab.net).

In addition to the established physiological measurements, two accelerometers (sampling rate 32 Hz) were used to record body (sensor located on the participant's chest) and hand-held console (sensor located in the backside of the console) movements. Acceleration data were integrated over one second and 3-dimensional axes were added together and rectified. From earlier studies we had reason to suspect that player movements might be associated with emotions during digital game play [48].

#### Questionnaire data

All questionnaires were administered in Finnish and delivered on paper. For background variables, we asked the participants to assess their previous experience of the game and the game console device with 6-point scales, from “Never played before” to “Played more than 50 times”. Given that the question related to the game device did not show any significant differences between genders when tested in the covariate analysis (see section Data analysis), it was discarded from our analyses. Trait questionnaire for Behavioral Inhibition and Activation System sensitivities (BIS and BAS) were used, as these are related to the propensity for negative and positive emotions due to punishment and reward [49].

Emotions were assessed by self-report measurements after both playing periods. Pleasure and Arousal scales from visual Self-Assessment Manikins (SAM; [50]), and shortened versions of Hostility (consisting of items “angry” and “hostile”), Fear (“frightened” and “nervous”), Joviality (“enthusiastic”, “excited”, and “delighted”), Serenity (“calm” and “relaxed”), and Sadness (“sad” and “downhearted”) subscales from Positive and Negative Affect Scale (PANAS; [51]) were administered. The Hostility and Fear subscales represented high-arousal negative (high and low dominance), Joviality high-arousal positive, and Serenity and Sadness low-arousal positive and negative affect, respectively. We found that the variance for the negative affect scales was smaller, and that the distribution of Hostility skewed strongly towards minimum value.

In addition, the Social Presence module, an earlier version of Social Presence in Gaming Questionnaire or SPGQ [52], was used (17 items). This questionnaire evaluates the following aspects of inter-player involvement and awareness: Behavioral Involvement (e.g., “What the other did affected what I did” and “What I did affected what the other did”), Empathy (e.g., “When the other was happy, I was happy” and vice versa), and Negative Feelings (e.g., “I tended to ignore the other”).

To examine potential confounds, Engagement and Spatial Presence subscales from the ITC-Sense of Presence Inventory with 27 items (ITC-SOPI; [53]) were administered, in addition to single items on 7-point scale to assess the anticipated threat, anticipated and experienced stressfulness and success (“How successful did you think you were in the previous game?”), and experienced performance (“How well did you perform, compared to your partner, in the previous game?”), mental struggling (“How much did you struggle to perform well in the previous game?”) and interest in the game, both before (anticipated stress, threat, and success) and after the play period. The anticipated assessments were used as baseline scores for the experienced assessments, except for anticipated threat which was used as a threat appraisal assessment.

#### Discarded data

Following the recommendation of Simmons, Nelson, and Simonsohn [54], we report that other self-rating data (four trait questionnaires and one state questionnaire, and an experimental game experience questionnaire) and respiratory activity data were collected as a part of our routine experimental paradigm but were not included in the present report, because they were not relevant for the present hypotheses.

#### Data processing and analysis

Average physiological signals during the baseline and each of the playing periods were calculated for each participant. Logarithmic transformations were conducted for facial EMG, SCL, and acceleration data to normalize the distributions. The data were analyzed with Linear Mixed Models procedure with maximum likelihood estimation in SPSS 20, due to the requirements of dyadic and repeated structure of the data [55]. Playing period was specified as the repeated variable, and first-order autoregressive model was specified as the covariance structure for the residuals. The members of the dyad were not interchangeable (the condition conducted in home environment was one participant's home but not the other's), so statistical indistinguishability (see [55]) was tested to find out whether the model should take this into account by treating the members as different. Because no differences were found, the members could be treated as indistinguishable, and we defined Dyad×Participant as the subject for the repeated term. The nonindependence of participants within dyads was initially taken into account by including random intercepts with Dyad as subject variable into the model [55]; however, this random variable was dropped because its effect was not significant in any of our analyses and it prevented some models from converging.

We tested our hypotheses using a basic statistical model, which included mode (cooperation or competition), gender, and mode×gender interaction as independent variables. Unless mentioned otherwise, all of our reported are based on this basic model. In addition, we also ran expanded covariate models to check for the effects of potential confounds, as suggested by Simmons and others [54]. This model included the following variables as covariates: environment (home or laboratory), interaction for environment×host (which participant's home the home environment was), the order of modes (competition or cooperation first), the order of environments (home or laboratory first), baseline value of the dependent variable (when available), and previous experience with the game. The last item was included in the confound model because our preliminary analyses indicated that males had more experience with the game than females (*p*<0.001). Any other of the tested potential confounds (anticipated threat, or experienced engagement, spatial presence, interest, success, performance, or struggle) did not differ significantly between the genders, *p*s>.1, and were hence not included in the covariate model.

Given the large number of variables studied in the present investigation, we used False Discovery Rate correction [56] for the significance thresholds to control for the inflated possibility of false positives. FDR of 5% resulted in threshold of .025 (*p*-values larger than that were declared non-significant).

### Results and Discussion

Results from the basic model analyses for all physiological variables are presented in Table 1, and for emotion self-report variables in Table 2. For the reader's convenience, we report the non-essential results, for body and console movement and social presence questionnaires, in Appendix S1 (Tables S1 and S2 in Appendix S1).

**Table 1:** Experiment 1 Linear Mixed Models for Physiological Dependent Variables.

	Estimated Marginal Means (*SE*)				
Model Variables	1	2	*df*	*F*	*p*
Zygomaticus Major EMG activity (ln[µV])					
Mode	2.907 (0.088)	3.175 (0.087)	1,134.128	38.647	**<.001**
Gender	3.158 (0.104)	2.924 (0.134)	1,50.566	1.919	0.172
Mode×Gender	2.907 (0.107)	3.410 (0.107)	1,134.128	29.411	**<.001**
	2.907 (0.139)	2.941 (0.137)			
Corrugator Supercilii EMG activity (ln[µV])					
Mode	2.594 (0.101)	2.583 (0.101)	1,136.477	0.195	0.66
Gender	2.539 (0.121)	2.638 (0.160)	1,46.814	0.241	0.626
Mode×Gender	2.553 (0.122)	2.525 (0.122)	1,136.477	0.377	0.54
	2.635 (0.162)	2.640 (0.161)			
Orbicularis Oculi EMG activity (ln[µV])					
Mode	3.066 (0.073)	3.237 (0.073)	1,136.213	26.073	**<.001**
Gender	3.083 (0.087)	3.220 (0.113)	1,51.245	0.921	0.342
Mode×Gender	2.913 (0.090)	3.253 (0.089)	1,136.213	25.467	**<.001**
	3.219 (0.116)	3.221 (0.115)			
Skin conducance level (ln[µS])					
Mode	1.301 (0.088)	1.311 (0.088)	1,129.997	0.406	0.525
Gender	1.285 (0.099)	1.327 (0.145)	1,44.022	0.058	0.81
Mode×Gender	1.263 (0.100)	1.307 (0.100)	1,129.997	4.836	0.03
	1.340 (0.146)	1.315 (0.146)			
Heart rate (BPM)					
Mode	74.523 (1.561)	75.866 (1.552)	1,130.594	10.286	**0.002**
Gender	74.500 (1.925)	75.889 (2.410)	1,46.876	0.203	0.655
Mode×Gender	73.208 (1.945)	75.792 (1.940)	1,130.594	8.761	**0.004**
	75.838 (2.441)	75.941 (2.424)			

*Note*. Intercept is left out as uninformative from all models. Means 1 and 2 correspond to Cooperative and Competitive for Mode, and Male and Female for Gender, respectively. For Mode×Gender interactions, the rows denote Male and Female, and the columns denote Cooperative and Competitive, in that order. The *p*-values significant after controlling the false discovery rate are bolded.

**Table 2:** Experiment 1 Linear Mixed Models for Self-Report Dependent Variables.

	Estimated Marginal Means (*SE*)				
Model Variables	1	2	*df*	*F*	*p*
PANAS-X Joviality					
Mode	4.795 (0.125)	5.004 (0.123)	1,129.863	3.033	0.084
Gender	4.885 (0.133)	4.914 (0.172)	1,59.348	0.019	0.892
Mode×Gender	4.612 (0.153)	5.158 (0.151)	1,129.863	7.946	**0.006**
	4.979 (0.198)	4.850 (0.195)			
PANAS-X Hostility					
Mode	1.752 (0.110)	1.765 (0.109)	1,123.946	0.016	0.899
Gender	1.695 (0.118)	1.822 (0.152)	1,53.679	0.435	0.512
Mode×Gender	1.678 (0.135)	1.711 (0.133)	1,123.946	0.034	0.853
	1.825 (0.174)	1.819 (0.172)			
PANAS-X Fear					
Mode	2.175 (0.100)	2.389 (0.099)	1,123.779	6.436	**0.012**
Gender	2.367 (0.110)	2.197 (0.142)	1,52.106	0.896	0.348
Mode×Gender	2.224 (0.123)	2.510 (0.121)	1,123.779	0.714	0.4
	2.125 (0.158)	2.268 (0.156)			
PANAS-X Serenity					
Mode	3.927 (0.129)	3.526 (0.127)	1,125.7	13.884	**<.001**
Gender	4.029 (0.143)	3.424 (0.184)	1,53.587	6.736	**0.012**
Mode×Gender	4.292 (0.158)	3.765 (0.156)	1,125.7	1.374	0.243
	3.561 (0.204)	3.286 (0.201)			
PANAS-X Sadness					
Mode	3.733 (0.071)	3.527 (0.070)	1,127.737	10.637	**0.001**
Gender	3.683 (0.078)	3.577 (0.100)	1,56.358	0.692	0.409
Mode×Gender	3.799 (0.087)	3.566 (0.086)	1,127.737	0.181	0.671
	3.666 (0.113)	3.488 (0.111)			
SAM Valence					
Mode	7.086 (0.147)	7.270 (0.145)	1,128.246	1.316	0.253
Gender	7.225 (0.153)	7.131 (0.190)	1,59.363	0.148	0.701
Mode×Gender	6.903 (0.184)	7.547 (0.181)	1,128.246	8.166	**0.005**
	7.269 (0.230)	6.994 (0.226)			
SAM Arousal					
Mode	6.188 (0.154)	6.366 (0.152)	1,124.389	1.282	0.26
Gender	6.386 (0.164)	6.168 (0.205)	1,55.956	0.69	0.41
Mode×Gender	6.050 (0.193)	6.722 (0.190)	1,124.389	9.798	**0.002**
	6.326 (0.241)	6.011 (0.237)			

*Note*. Intercept is left out as uninformative from all models. Means 1 and 2 correspond Cooperative and Competitive for Mode, and Male and Female for Gender, respectively. For Mode×Gender interactions, the rows denote Male and Female, and columns denote Cooperative and Competitive, in that order. The *p*-values significant after controlling the false discovery rate are bolded.

#### Hypothesis 1: higher positive emotion in competition for males

**Figure 1:**
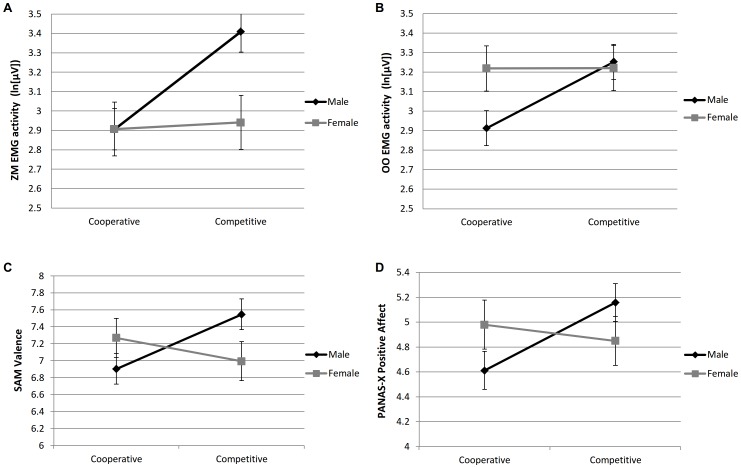
Gender differences in zygomaticus major (top left panel) and orbicularis oculi (top right) EMG activity, both associated with positive emotion, and self-reported pleasantness (bottom left) and positive affect (bottom right), across cooperative and competitive modes (Experiment 1). The error bars represent standard errors.

Hypothesis 1 was supported by the physiological measurements and self-reports: the significant interaction of mode and gender for both ZM and OO EMG activity (Figure 1, top panels) showed that males exhibited more positive emotion during competition than during cooperation (both *p*s<.001, see Table 1), and that they reported higher Joviality and SAM Valence in the competitive mode than in the cooperative mode, (*p*s = .006 and .005, Figure 1, bottom panels). See Table S1 in Appendix S1, for body and console movement results, and Table S2 in Appendix S1, for social presence results.

#### Hypothesis 2: higher positive emotion in cooperation for females

We found no support for Hypothesis 2, as there were no significant differences between cooperation and competition for females in ZM and OO EMG activity (Figure 1, top panels) or PANAS Joviality or SAM Valence (Figure 1, bottom panels).

#### Hypothesis 3: higher arousal in competition for both genders

**Figure 2:**
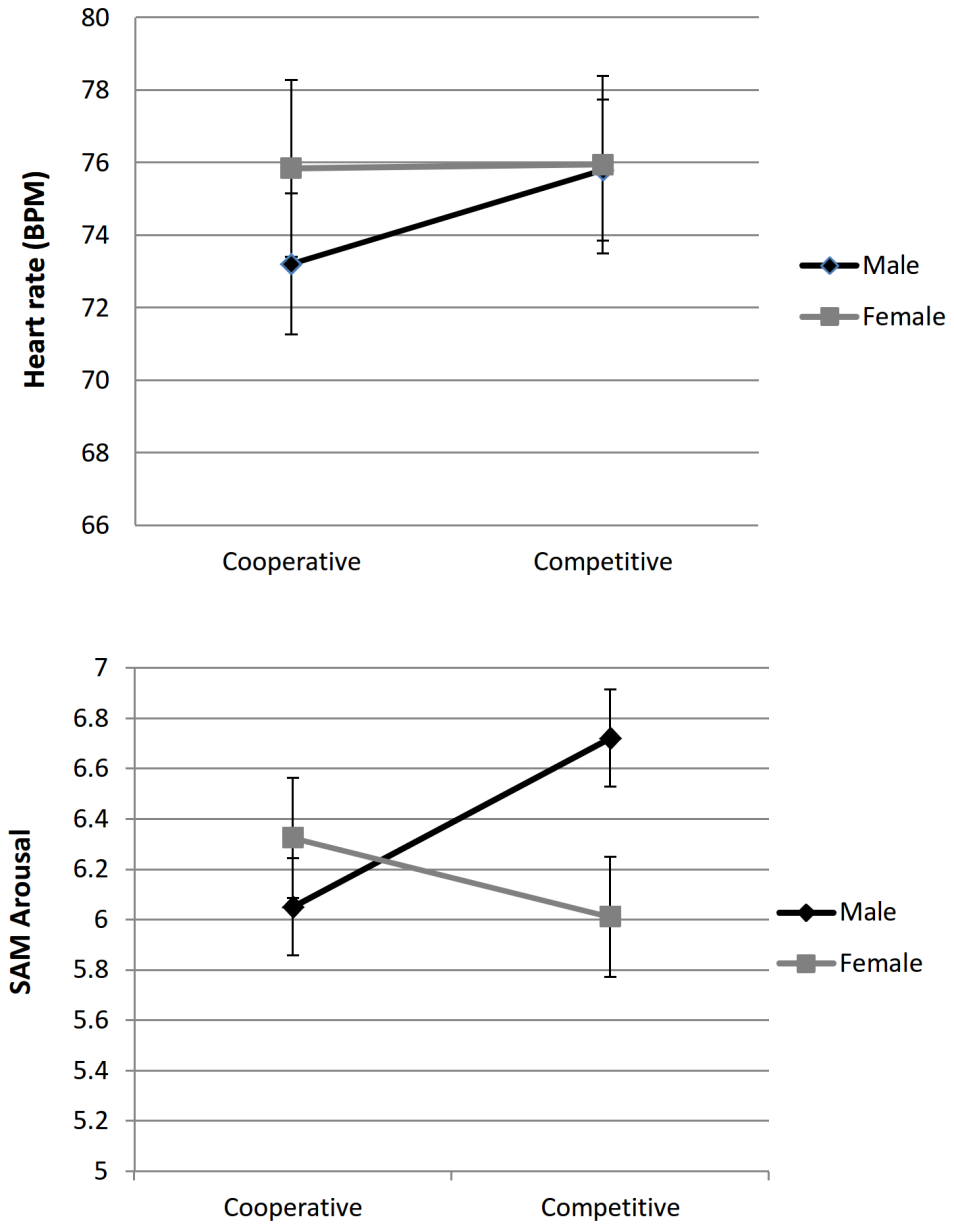
Gender differences in heart rate (left panel), associated with emotional arousal, and self-reported arousal (right panel), across cooperative and competitive modes (Experiment 1). The error bars represent standard errors.

HR was higher in the competitive versus cooperative mode (*p* = .002), but a significant interaction between mode and gender (*p* = .004) revealed that this effect was present only for males (Figure 2, top panel). SCL showed no significant main effect for mode, but instead an interaction similar to HR with a decrease for females and an increase for males from cooperative to competitive mode, although the interaction was not significant after FDR correction (*p* = .030). In self-reports, SAM Arousal showed the same interaction pattern where males reported higher and females (slightly) lower arousal during competition than during cooperation, (*p* = .002, Figure 2, bottom panel); but again the main effect for the mode was non-significant. In addition, self-reports related to low-arousal discrete emotions suggested that cooperation may have had a calming effect on both males and females: both genders reported feeling less Fear (*p* = .012), and more Serenity and Sadness (*p*<.001 and *p* = .001) during cooperation than competition.

Contrary to our Hypothesis 3, these results hence suggest that competition versus cooperation elicited opposite rather than similar effects between the genders (higher arousal in males and lower arousal in females).

#### Research question 1: is there more or less negative emotion in competition?

We found no association between mode and CS EMG activity or PANAS Hostility (*p*s>.1), and although participants reported experiencing more Fear and less Serenity and Sadness during competition (see above), this can be interpreted as an effect of arousal rather than negative emotion.

We conclude that competition does not seem to be experienced negatively as such (but see Table S2 in Appendix S1), at least in the intrinsically motivated competition used in this experiment.

#### Covariate models

The results for mode, gender, and their interaction did not notably change for any of the dependent variables for the covariate models that included environment, environment×host interaction, the order of modes and environments, and the baseline of the dependent. The only notable difference was that the weak interaction effect for SCL was even weaker (p<.042) when the covariates were included in the model.

Previous experience, despite of being reported significantly different for males and females, revealed no significant association with any of the dependent variables, in main effect nor in interaction between previous experience and gender.

Baseline was extremely significantly associated (all *p*s<.001, except *p* = .003 for Fear and .034 for Sadness) with almost all the variables (except for Serenity, n,s; for SAM there was no baseline), showing that there were significant individual differences between the participants. However, including these baseline values in our analyses did not change any of the above significant findings.

Order of mode and the environment did have a significant positive effect on the positive affect measures, i.e. ZM and OO EMG activity (*p*s = .004 and .008 for ZM, .006 and .016 for OO), and SAM Valence (*p*s = .017, and .018), as well as body and console movement (*p* = .004 and .025 for order of mode, but n.s. for environment). This shows that the later experimental periods elicited higher positive affect compared to earlier periods, and that home was experienced more positively than the laboratory, but that these effects did not affect the interactions between gender and mode (see above). Order of mode showed the same pattern as the positive emotions for HR (*p* = .023) but not for SCL (*p*s>.1). It seems plausible that the later experimental periods and periods at home have felt more comfortable and relaxed, allowing higher positive affect. This could also have lead to an effect for environment in Hostility, higher at the laboratory (*M* = 1.936, *SE* = 0.099) compared to home (*M* = 1.499, *SE* = 0.100, *t*(166,172) = 2.844, *p* = .005).

## Experiment 2

In the second experiment we sought to test the findings from the first experiment with a different game, while addressing some concerns that might have affected its results. First, it is possible that the type of game (quick action) might have required a constant high level of arousal, effectively masking any arousal differences between the modes (Hypothesis 3), and possibly making the game more likable to males than females [57]. Consequently, we chose a new stimulus game that would not require constant quick action. A turn-based game would allow the participants to play the game on their own pace, which should allow for lower arousal levels and thereby provide better data on arousal differences in different conditions. This might also affect which processes are dominant on heart rate: if HR responded to arousal in a fast-paced game, it might be that during a less arousing game HR responds more to the attention component [58].

Second, it is possible that our dichotomy between competition and cooperation was too absolute, given that some activities may at the same time contain both coincidental and opposing goals between the participating individuals (cf. [59], pp. 254–256). For example, some sports activities combining both competition and cooperation have been reported to be more enjoyable than sports activities containing only either one of them [22]. Thus, we added a condition with both coincidental and opposing individual goals to the present study. Specifically, participants were assigned to the same team so that they scored wins or losses together against the computer-controlled team; however, at the same time they also competed individually for higher score within the team.

Third, we did not specifically (besides with subjective measures) control for the players' varying performance levels in the first experiment. In the second experiment, we recorded the personal and team victories and losses, and included them in our analyses.

### Methods

#### Participants

The participants were 100 Finnish university students recruited in 50 same-gender dyads (21 female dyads). Due to technical difficulties, 9 of the dyads had to be removed from the physiological dataset, which resulted in 82 participants in 41 dyads (16 female), with age ranging from 18 to 32 (M = 23.0, SD = 3.2 years). The participants received information and compensation and signed an informed consent form similarly as in Experiment 1.

#### Materials

The stimulus game was Hedgewars (http://hedgewars.org), an open-source clone of a popular commercial game Worms (Team 17). Hedgewars is a turn-based artillery game, which features two or more teams of cartoon-like characters on a two-dimensional map. The game's goal was to eliminate all other teams' characters by using various cartoonish weapons, either by reducing their health to zero or by managing to knock them into the water. Players had 45 seconds per turn to move a character (only one was moved at a time), choose a weapon, and shoot by adjusting the power and angle of the ballistic shot. Turn order was randomized. During the course of the game, players were able to gain new weapons by picking up boxes that landed on the map at constant intervals. Most of the game's weapons were turned off, however, so that the more experienced gamers would not have a significant edge over the less experienced ones, and to reduce the number of possible actions to a more manageable level.

#### Procedure

After the practice session and a 5-min baseline recording, the two players in each dyad sat next to each other in the laboratory and played the game on the same computer, sharing mouse and keyboard in turns to control the game. Separate computers on side desks were used for answering self-report questionnaires both before and after each experimental period.

Each dyad played the game in four different conditions, which were presented in a randomized order. The conditions were: (1) playing in the same team against a computer-controlled team (cooperation); (2) playing in the same team against a computer-controlled team with equal number of characters, while competing against each other for the higher score (cooperation-competition); (3) playing in different teams against each other, with equal number of computer-controlled characters in both teams (competition); and (4) playing in different teams against each other, without computer-controlled team members (competition without computer). Computer-controlled team characters were included in condition 3 in order to maintain both similar team sizes (six characters) and numbers of player-controlled characters (three characters) as in conditions 1 and 2. Computer-controlled team members were removed from condition 4 to control for the possible effects of computer-controlled within-team characters, keeping the player-controlled characters constant (resulting in three vs. three characters in condition 4, as opposed to six vs. six in other conditions). Of these, conditions 1 and 3 were equivalent to the cooperation and competition modes of Experiment 1, respectively.

When asked, 17 out of 82 participants (13.94%) reported erroneously after condition 2 that they had been playing condition 1, in contrast to only one or two mistakes in the other conditions. Apparently, the difference between conditions 1 and 2 was not as clear as differences between the other conditions, despite the fact that the playing condition was explicitly stated to the participants before the beginning of each game. We removed these mistaken playing periods from our data.

#### Physiological data acquisition

Physiological data was acquired the same way as in Experiment 1, with the following exceptions to the processing of EDA and ECG signals. EDA signal was downsampled to 4 Hz and smoothed using Ledalab (V.3.2.5) toolbox for Matlab, and divided into phasic and tonic components using the non-negative deconvolution method [60]. The tonic component was averaged over each play period to skin conductance level (SCL). The advantage of this method is that because phasic variation is removed from the signal, it produces a less noisy measurement of the tonic component than direct averaging from the original signal. ECG signal was analyzed using the ECGLab toolbox for Matlab. R-peaks were identified from the original 512 Hz series and corrected for ectopic beats. Interbeat interval (IBI) time series was obtained, and HR was calculated as an inverse of IBI (i.e., 60000/IBI).

#### Questionnaires

All questionnaires were administered in Finnish on a computer. In pre-experimental questionnaires we asked the participants to evaluate their previous experience with Hedgewars game (the present game), Worms game (the game HW is a clone of), and artillery games in general. Because the previous experience with Worms differentiated the genders best, only this item was retained for further analyses. Similarly as in Experiment 1, pre- and post-condition questionnaires included PANAS-X Joviality, Fear, and Hostility scales (but not Serenity and Sadness); SAM Valence and Arousal scales, now added with Dominance scale; separate single items assessing anticipated threat, and anticipated and experienced struggle, success and stressfulness (anticipated assessments were again used as baseline scores, where applicable); and the Social Presence in Games Questionnaire; and BIS and BAS sensitivity scales as a trait questionnaire. In addition, we added the 5-point perceived comprehension scale from Social Presence Inventory (with items such as “My mood affected the mood of the other”, both for me affecting the other and the other affecting me; [61]), shortened Attentiveness scale (2 items) from PANAS-X, and separate items for experienced frustration, being entertained, and intensity. We also had an open question to provide the participants a way to comment if something specific affected their answers.

#### Discarded data

In addition to the reported variables, we administered four state and eight trait questionnaires not relevant for the present study. Furthermore, we calculated various heart rate variability indices and compliance scores for all the physiological signals, which have been reported elsewhere [62], [63]. We also measured body movement, but due to techical difficulties this data had to be discarded.

#### Data analysis

Data analysis was similar to Experiment 1, with minor modifications. In the basic model, instead of two-level mode, we used a four-level condition variable to test for differences between cooperation and competition, the final factors being gender, condition, and gender×condition interaction. The nonindependence of participants within dyads was taken into account by including random intercepts for dyads into the models of physiological (but not self-report) data. Unlike in the first experiment, variances for these intercepts were different from zero and were retained. The exception was the model with HR as dependent, for which the random effect was removed.

Similarly to the first experiment, we used a separate model to test for the effects of potential confounds. This model included the following covariate effects: order of conditions, baseline value of the dependent variable, previous experience with Worms game, game period length, and game scores. Game length was included because—unlike in Experiment 1—the playing time was not fixed. Game scores were included as a covariate because our preliminary analyses revealed that males were more successful against the computer opponents than females. Males also reported higher experienced performance than females (*M* = 3.41 for males and 2.98 for females, *t*(81.301) = 2.612, *p* = .011), but we discarded this variable from the covariate model as redundant after including game score. Other variables of interest were not significantly different between males and females in our preliminary analyses (*p*s>.05). Finally, FDR was calculated as in Experiment 1, resulting in threshold of 0.0178.

### Results and Discussion

The results for all physiological variables are presented in Table 3, and for self-report variables in Table 4. Similarly as in Experiment 1, social presence results are available in Table S3 in Appendix S1.

**Table 3:** Linear Mixed Models for the Physiological Dependent Variables in Experiment 2.

	Estimated Marginal Means (*SE*)						
Model Variables	1	2	3	4	*df*	*F*	*p*
Zygomaticus Major EMG activity (ln[µV])							
Condition	1.46 (0.068)	1.491 (0.069)	1.699 (0.068)	1.721 (0.068)	3,206.116	29.538	**<.001**
Gender	1.769 (0.081)	1.417 (0.101)			1,40.898	7.364	**0.01**
Condition×Gender	1.56 (0.085)	1.668 (0.087)	1.928 (0.085)	1.918 (0.085)	3,206.116	5.26	**0.002**
	1.36 (0.106)	1.314 (0.107)	1.469 (0.106)	1.524 (0.106)			
Corrugator Supercilii EMG activity (ln[µV])							
Condition	1.063 (0.027)	1.074 (0.028)	1.021 (0.027)	0.991 (0.027)	3,211.781	13.552	**<.001**
Gender	0.984 (0.032)	1.091 (0.04)			1,41.149	4.303	0.044
Condition×Gender	1.008 (0.034)	1.007 (0.035)	0.959 (0.034)	0.962 (0.034)	3,211.781	2.619	0.052
	1.119 (0.042)	1.142 (0.043)	1.082 (0.042)	1.021 (0.042)			
Orbicularis Oculi EMG activity (ln[µV])							
Condition	1.606 (0.05)	1.626 (0.051)	1.817 (0.05)	1.828 (0.05)	3,208.14	34.546	**<.001**
Gender	1.854 (0.06)	1.585 (0.074)			1,40.977	7.952	**0.007**
Condition×Gender	1.69 (0.063)	1.75 (0.065)	1.989 (0.063)	1.985 (0.063)	3,208.14	3.994	**0.009**
	1.523 (0.078)	1.502 (0.08)	1.646 (0.078)	1.67 (0.078)			
Skin conductance level (ln[µS])							
Condition	2.204 (0.048)	2.205 (0.049)	2.227 (0.048)	2.231 (0.048)	3,222.169	3.317	0.021
Gender	2.244 (0.06)	2.19 (0.075)			1,41.061	0.318	0.576
Condition×Gender	2.24 (0.061)	2.243 (0.061)	2.243 (0.061)	2.251 (0.061)	3,222.169	1.96	0.121
	2.169 (0.076)	2.167 (0.076)	2.212 (0.076)	2.212 (0.076)			
Heart rate (BPM)^a^							
Condition	74.02 (0.96)	74.079 (0.969)	74.581 (0.958)	75.747 (0.957)	3,220.154	10.354	**<.001**
Gender	74.438 (1.174)	74.776 (1.464)			1,82.078	0.032	0.858
Condition×Gender	73.311 (1.203)	73.778 (1.216)	74.77 (1.198)	75.893 (1.197)	3,220.154	3.029	0.03
	74.729 (1.497)	74.381 (1.509)	74.392 (1.497)	75.601 (1.494)			

*Note*. Intercept is left out as uninformative from all models. Means 1 through 4 correspond to Cooperative, Cooperative and Competitive, Competitive, and Competitive Without Computer. Means 1 and 2 correspond to Male and Female for Gender. For Gender×Condition interactions, the rows denote Male and Female and the columns denote the Condition. The *p*-values significant after controlling the false discovery rate are bolded.

a Heart rate run with independent members.

**Table 4:** Linear Mixed Models for Self-Report Dependent Variables in Experiment 2.

	Estimated Marginal Means (*SE*)						
Model Variables	1	2	3	4	*df*	*F*	*p*
PANAS Joviality							
Condition	3.665 (0.089)	3.589 (0.094)	3.385 (0.089)	3.499 (0.089)	3,221.971	4.838	**0.003**
Gender	3.592 (0.096)	3.477 (0.117)			1,79.724	0.581	0.448
Condition×Gender	3.745 (0.113)	3.566 (0.121)	3.505 (0.112)	3.553 (0.113)	3,221.971	1.043	0.374
	3.586 (0.137)	3.612 (0.143)	3.266 (0.137)	3.445 (0.137)			
PANAS Hostility							
Condition	1.297 (0.073)	1.297 (0.08)	1.508 (0.072)	1.34 (0.073)	3,225.987	2.564	0.056
Gender	1.273 (0.063)	1.448 (0.077)			1,81.384	3.089	0.083
Condition×Gender	1.188 (0.092)	1.209 (0.104)	1.375 (0.092)	1.32 (0.092)	3,225.987	0.622	0.602
	1.406 (0.112)	1.385 (0.121)	1.641 (0.112)	1.359 (0.112)			
PANAS Fear							
Condition	1.451 (0.061)	1.445 (0.066)	1.422 (0.061)	1.48 (0.061)	3,221.643	0.283	0.837
Gender	1.391 (0.061)	1.508 (0.074)			1,78.575	1.469	0.229
Condition×Gender	1.339 (0.078)	1.406 (0.086)	1.375 (0.078)	1.444 (0.078)	3,221.643	0.629	0.597
	1.562 (0.095)	1.483 (0.101)	1.469 (0.095)	1.516 (0.095)			
SAM Valence							
Condition	6.982 (0.174)	7.03 (0.191)	6.417 (0.173)	6.709 (0.174)	3,224.437	3.364	0.019
Gender	7.034 (0.149)	6.535 (0.18)			1,79.531	4.543	0.036
Condition×Gender	7.526 (0.221)	7.077 (0.249)	6.646 (0.219)	6.886 (0.221)	3,224.437	1.836	0.141
	6.437 (0.268)	6.983 (0.289)	6.187 (0.268)	6.531 (0.268)			
SAM Arousal							
Condition	6.513 (0.16)	6.331 (0.171)	6.063 (0.159)	6.323 (0.16)	3,222.835	2.657	0.049
Gender	6.306 (0.161)	6.309 (0.196)			1,79.892	0	0.99
Condition×Gender	6.464 (0.203)	6.207 (0.222)	6.125 (0.202)	6.427 (0.203)	3,222.835	0.74	0.529
	6.563 (0.247)	6.454 (0.261)	6 (0.247)	6.219 (0.247)			
SAM Dominance							
Condition	6.546 (0.178)	6.573 (0.196)	5.177 (0.177)	6.443 (0.178)	3,224.032	17.802	**<.001**
Gender	6.398 (0.146)	5.972 (0.177)			1,78.522	3.434	0.068
Condition×Gender	7.092 (0.226)	6.882 (0.256)	5.167 (0.224)	6.449 (0.226)	3,224.032	2.809	0.04
	6 (0.274)	6.264 (0.296)	5.188 (0.274)	6.438 (0.274)			

*Note*. Intercept is left out as uninformative from all models. Means 1 through 4 correspond to Cooperative, Cooperative and Competitive, Competitive, and Competitive Without Computer. Means 1 and 2 correspond to Male and Female for Gender. For Gender×Condition interactions, the rows denote Male and Female and the columns denote the Condition. Note that the figures here are from the basic models without the confounding game result as covariate. The *p*-values significant after controlling the false discovery rate are bolded.

#### Hypotheses 1 and 2: higher positive emotion in competition for males, and in cooperation for females; covariate models potentially explaining the differences to Experiment 1

**Figure 3:**
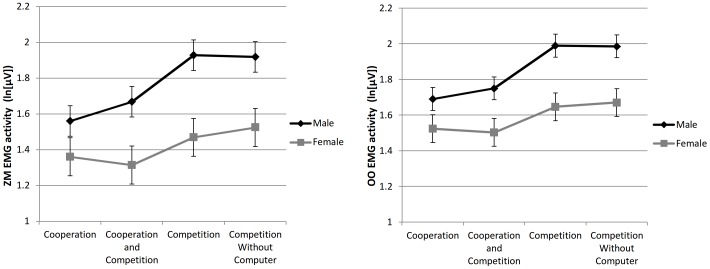
Gender differences in zygomaticus major (left panel) and orbicularis oculi (right panel) EMG activity, both associated with positive emotion, across the four conditions representing different modes of competitiveness (Experiment 2). The error bars represent standard errors.

Supporting Hypothesis 1, but not Hypothesis 2, significant interactions between condition and gender for ZM (*p* = .002) and OO EMG activities (*p* = .009; Figure 3, left and right panels, respectively) revealed that males, but to a lesser degree also females, had more positive emotional responses to competitive (conditions 3 and 4) than to cooperative (1 and 2) conditions (Table 3).

Self-reports, however, failed to support Hypotheses 1 and 2; that is, SAM Valence and PANAS Joviality showed no significant interactions between gender and condition (*p*s>.1; see Table 4). The main effect for Joviality (*p* = .003, but for Valence, *p* = .019, n.s.) suggests that actually cooperative conditions were reported as more positive, both by males and females. However, the covariate models showed a strong positive effect for game result (*F*(2, 223.861) = 11.270, *p*<.001 for Joviality, and *F*(2, 244.533) = 30.633, *p*<.001 for Valence), indicating that winning the game resulted in higher self-reported positive emotions (*M*s = 3.403, 3.545, and 3.727 [Joviality], and 5.986, 6.737, and 7.350 [Valence] for game lost, game won by team member, and game won, respectively). It seems likely that emotional self-reports given after the game reflected emotional reactions to knowing the game's final score more than emotional reactions that were actually experienced during the game. Therefore, even if there were differences between genders across conditions, they would be confounded by the strong response to victory or defeat (cf. [64]–[66]). Unfortunately this limits the usefulness of these self-reports. It should be noted that in Experiment 1, although we did not control for victories and losses (and did not explicitly present the results to the participants as we did here), the participants played several matches during a fixed-length period and may have both won and lost during the game, thus on average probably reducing this effect. Physiological measurements, on the other hand, were collected during the game, while the winner was still unclear, and therefore reflected the emotions experienced *during* condition, instead of reflecting the response to game result afterwards.

In an attempt to control for the effect of game result, we ran post-hoc LMMs for Valence and Joviality separately for game won and lost. It was found that Joviality followed the pattern of physiological positive emotion measures when the game was won (but not when it was lost), although with power suffering from lower *n* the interaction was not significant (*p* = .034, main effect for condition at *p* = .011). Valence showed no significant associations (*p* = .1 for interaction, *p* = .031 for condition main effect when game was won). These might point to similar pattern to in the first experiment after victory but with no further means to distinguish the effect of the game result and the experience during play, this remains somewhat speculative.

#### Hypothesis 3: higher arousal in competition for both genders

As in Experiment 1, HR was strongly associated with conditions (main effect *p*<.001), and the increase in competitive conditions was somewhat stronger for males (*p* = .030 for the interaction), however the effect was nonsignificant when corrected for FDR. SCL showed no main effect for condition (*p* = .021, n.s.), and no interaction (*p*>.1). Although the game result did not have a significant effect on either physiological variable in the covariate model (*p*s>.1), the effects strengthened for HR (*p*<.001 for main effect and *p* = .012 for interaction), but weakened for SCL.

SAM Arousal showed a non-significant decrease (not increase) in competitive conditions in both basic (*p* = .049) and covariate (*p* = .033) models. (Game result was also only weakly associated with Arousal (*p* = .023) in the covariate model.)

The conclusion remains that there is no support for Hypothesis 3. Instead of an action game as in Experiment 1, we used a slower-paced turn-based game here. If the HR effect in Experiment 1 was simply a result of bodily activation and not the competition per se, it should have disappeared here. Instead, HR repeated the pattern resembling the one by positive affect measures, different from SCL that supposedly reflects the arousal closer, but also different from self-reported arousal (although the effect of game result might have affected this). The alternatives remain that HR responds successfully to arousal from the manipulation while SCL for some reason does not, or that while no differences can be found in arousal, HR might be associated specifically with high-arousal positive affect, seeing as how it follows the same pattern as ZM and OO EMG activity.

It has also been found that increasing tonic HR is related to decreasing attention (see [39]). For this, we ran a separate LMM with PANAS Attentiveness as dependent variable, but as the interaction was nonsignificant, it suggests that this explanation for HR was not valid here.

#### Research question 1: is there more or less negative emotion in competition?

Unlike in Experiment 1, the model for CS EMG activity revealed an effect for condition (*p*<.001), showing higher activity in cooperative – not competitive – conditions. This rather points to reciprocal activation of positive and negative emotions, not to uncoupled activation as did the results of Experiment 1, given that self-reported Hostility and Fear did not corroborate these results.

#### Post-hoc analyses of potential explanators for gender differences

To investigate the gender differences more in depth, some variables measured as potential confounds were examined separately in the analysis phase. Control questions included items about how stressed the participants were about the upcoming match and how threatening they considered it, both related to a potential explanation of risk-aversiveness [2], [6], by which females tend to view a competition as a threat rather than a challenge and therefore react with more stress; and an item about how well they anticipated they would perform during the upcoming match, related to a potential explanatory variable of self-confidence (regardless of skills or performance, males tend to believe they will perform well; [3], [6]). Furthermore, the trait Behavioral Inhibition System (BIS) and Behavioral Activation System (BAS) sensitivity between genders was also compared, because the former is associated with sensitivity to anticipated negative and the latter with anticipated positive emotions [49]. Post-hoc analyses were conducted for these variables from both experiments, with gender, mode/condition, and their interaction as predictors, and the repeated effect defined as described for the basic analyses (except for BIS and BAS sensitivity, which were single trait scores and therefore could not have a repeated effect).

The results can be found from Tables S4 and S5 in Appendix S1. In short, we found that females tended to negligibly anticipate more stress (*p* = .139 in Experiment 1, *p* = .023 in Experiment 2), possibly associated with the gender difference in BIS sensitivity (*p* = .001), but no difference in anticipated threat, playfulness, or BAS measures, and the higher self-confidence in males was explained by their previous experience with the game.

#### Covariate models

As explained above, game result did not had no significant effects on any of the physiological variables, but it exerted a strong effect on most self-reports (with the exception of Fear, *p*>.2). This supports the interpretation that self-reports reflected the game result more than actual affect during play, while physiological signals were unaffected by this.

Of the other covariates, period length was negatively associated with OO (*p*<.001; *p* = .030 for ZM) EMG activity, suggesting that the shorter (with more effective – and possibly more entertaining – single shots) games were experienced more positively than the lengthier ones. Order of period was positively associated with CS EMG activity (*p* = .006), and negatively with SCL and HR, (*p*<.001 and *p* = .005), but not to ZM or OO EMG activity as in Experiment 1. Baseline values were strongly associated with the physiological dependent variables (and self-reported Joviality and Fear) with all *p*s≤.003, again illustrating that individual differences matter, but they did not change the general results.

#### Other findings

SAM Dominance showed a significant effect for condition interaction (*p*<.001), both genders reporting clearly lower ratings during the competitive condition where the participant was teamed with a computer player. This pattern looks similar to patterns in Joviality, Hostility (inversely), Valence, Arousal, and Behavioral Involvement and Perceived Comprehension (the social presence results in Table S3 in Appendix S1), which seems too regular to be coincidence. Because more than one participant commented for the open question that the computer team mate “made stupid choices that I felt I could not affect” (such as who to target and the weapon choices), this might have been seen as hindering the participants' own goals due to their different (and invisible) logic, possibly affecting emotion and social presence. This effect was probably not explained by the computer players' poorer performance; we tested this with a post-hoc analysis by using a basic LMM with the single item “I felt frustrated” as the dependent variable and by controlling the game result as a random variable, but found no main effect for condition (*p*>.5) and no interaction (*p* = .092). Apparently, these results may have been caused by the observation that the reasons for the computer players' choices were undecipherable, and this might lead to reduced positive emotions, dominance, and self-reported social presence. This interpretation is related to findings that computer player's presence adversely affects the physiological assessment of social presence (see [62], [63]).

## General Discussion

Earlier research suggests that males prefer competition and females prefer cooperation. For instance, in economic experiments [6] and cognitive tasks [2], [3] males choose competition (over non-competitive scoring) more often than females; in sports and games male motivations are more oriented to competition than female motivations [8], [28]; and it has been even suggested that females are biologically wired to be more cooperative [9] (see also [17]). Because preferences are based on emotions [29] it would be expected that these preferences are mirrored by similar differences in emotions. Our two studies examined the differences between male and female emotional responses to competition and cooperation during and after (intrinsically motivated) game play, controlling for various confounding factors. In both experiments, we found strong support for Hypothesis 1 that males would exhibit more positive emotions during competitive than cooperative game play, but no support for Hypothesis 2 that females would show more positive emotions during cooperative game play. The physiological evidence based on both zygomatic and orbicularis EMG responses (associated with positive emotions; see [34], [67], [68]; for studies in game context, see e.g., [65], [69]) was strong, revealing the same positive association with competitive game play for males and a lack of difference between modes for females in both experiments. Self-reported positive emotions corroborated this evidence in Experiment 1. In Experiment 2 the self-reported positive emotions followed the same pattern for males only to a limited extent, but the explicit way of declaring the winner after the game may have affected these results. We also found that these results held regardless of skills and previous experience with the game, perceived performance, the initial interest in the activity, and whether the participants won or not.

Although cooperation and competition are often discussed as polar opposites to each other (e.g., [10], [17], [70]), which leads to the assumption that not preferring competition indicates preference for cooperation, it is important to note that the specific evidence on female tendency for cooperation is much weaker than the evidence on male preference for competition. Actually, it has been a long-standing issue that despite the idea of female cooperativeness present in the gender roles (e.g., [71]), female self-perceptions (e.g., [11], [12]), and evolutionary psychological theories [7], [10], the experimental evidence has been hard to find and conflicting [72]–[74]. Furthermore, both reviews on the subject [6], [72] state that the occasional gender difference in cooperativeness seems to be very context sensitive. As the most research on the subject is from social dilemmas (such as dictator games) instead of real-life behavior, it is not clear whether any possible differences even could be generalized to another particular context—and the only previous study from gaming context [30] did not report gender effects for cooperation or competition. Our results support the view that, in addition to females not preferring cooperation over competition, females are not more cooperative than males. The between-subjects comparison of physiological responses is problematic because of large individual differences present in these measures [75], but the fact that zygomatic activity was overall higher in males while females are generally more prone to present that activity higher [41] suggests—along with self-reports—that this conclusion is not too far-fetched here. Whether this is generalizable to intrinsically motivated activities beyond simple digital games is a question left for future research.

The theoretical basis behind the gender difference in tendency to compete is, as stated, much better established than the evidence on differences in cooperation. Several mechanisms have been proposed in the literature, among others the females' higher risk-aversiveness and male overconfidence: the former refers to the female tendency to view a competition as a threat rather than a challenge, and the latter to results that, regardless of skills or performance, males tend to believe they will perform well [2], [3], [6]. We did not measure risk-aversiveness and self-confidence as such, but we did have control questions for potential confounds on how stressed the participants were about the upcoming match, how threatening they considered it, and how well they anticipated they would perform during the upcoming match. Our post-hoc investigation revealed that there was no difference in anticipated threat, nor in anticipated success between genders—although the males did anticipate better success than females before the match (indicating higher self-confidence), this difference was explained by the difference in previous experience with the game. However, anticipated stress was a bit higher for females (in Experiment 2), and we also found higher Behavioral Inhibition System sensitivity in females—a measure that indexes the biological system behind responding to anxiety-relevant cues in environment [49]. While connection of stress and BIS sensitivity and the female tendency to higher BIS sensitivity scores have been reported earlier [76], [77], the evidence suggests that higher BIS sensitivity should be associated with more negative emotions (rather than less positive; see [44], for evidence for separability of positive and negative), yet our analyses of the physiological or self-reported negative emotions did not show a gender difference (RQ1). Hence, at least with our limited measures, gender differences in self-confidence did not receive support as an explanation for the gender differences in emotions, and similar threat appraisal results for males and females failed to support the other viable explanation based on risk-aversiveness in females. Although females reported more stress, which would be consistent with the risk-aversiveness explanation, there was no interaction effect on stress between gender and mode. While it is conceivable that a lower level of stress would make it easier to experience positive emotions, it is unlikely to be the sole source of the difference in positive emotions between genders.

Another possibility to explain the difference in responses to competition is provided by the framework of the self-determination theory [23], and the findings that positive and negative affect has a somewhat direct association with intrinsic and extrinsic motivation, respectively (in the context of sports, see [78]; for digital games, see [79]). It might be that—instead of e.g., higher reward-seeking behavior in males—the mechanism for both competitive preferences and consequent positive affect stems from the cultural gender expectations that perpetuate the higher male need for competence (e.g., [80]) and the needs satisfaction fulfilled by competition. For example, Gneezy, Leonard, and List [81] report how a matrilineal society in India with different gender roles shows the pattern of higher female preference for competition, and this would be also in line with the findings that masculine gender roles are associated with increasing intrinsic motivation in competition [25]. Further, the findings about male overconfidence (e.g., [3]) might be related to high perceived competence that comes with the gender expectations. While we assumed a game being played by people who like to play games an intrinsically motivated activity, the motivation is not a binary state, but a continuum, allowing differences in the level of intrinsic motivation (cf. [78]). Our results could therefore reflect the culturally higher male need for competence and fulfillment of that need by competition. Without explicit measurements, however, we do not know how the participants would have reported their needs or motivation, so this explanation remains conjectural.

Our Hypothesis 3 concerned higher arousal in competition, compared to cooperation, regardless of gender. The findings were conflicting. Heart rate, a signal often associated with arousal [33], was higher in competition for males but not for females in both experiments, an effect closely resembling to the one of positive affect measures. On the other hand, skin conductance level, the most widely used measure of physiological arousal [33], [39], [82], did not show a significant difference between the modes, and the self-reports supported HR in Experiment 1, but lacked any effect in Experiment 2. Given that the neural control of electrodermal activity is exclusively under sympathetic nervous system while the heart rate is innervated by both sympathetic and parasympathetic pathways, the skin conductance could be considered a more reliable index of arousal during complex stimuli [39]. On the other hand, electrodermal responses habituate quickly [82], which might lead to weakened differences in measures of tonic skin conductance level during longer experimental periods, though we have not found this to be a problem in our previous experiments. Another possibility comes from following the line of explanations about gender role expectations presented above. Given that the cardiovascular activity respond to gender-roles—whether the participants consider the task something their gender should be capable at [83],[84]—HR might show a sensitivity to specifically high-arousal positive affect in the context of competitive digital game (cf. [39]). HR results could therefore indicate that the participants considered winning in a digital game more relevant to males. Again, as we did not assess gender-role orientation and perceived ability, this remains speculative.

### 

#### Other Findings

Social presence was found to be linked to positive affect at least to some extent, which is not surprising per se [85], [86]. However, we also found that certain emotional states normally labeled as negative (such as schadenfreude and revengefulness) might be associated with positive affect. As they were experienced as part of the (playful) competitive setup, this might be a sign of so-called meta-emotions (cf. [21], [87]). Social presence also responded to the presence of computer-controlled characters, showing that the social presence might be reduced because of them (cf. [62], [63], for assessment of social presence with so-called physiological linkage),

There have been only preliminary indications what would be the association between body or hand movement and emotions [88], but according to our results they seem to be closely related with the cooperative-competitive manipulation. This might suggest that movement is related to positive emotions during competition, presumably as the participants move more when they are having fun with a friend, or perhaps to arousal, if results with heart rate can be interpreted as such. As the acceleration sensors are becoming more commonplace within consumer electronics, they might become an easy way to assess at least some aspects of emotions, if the association can be ascertained and replicated in the vastly different situations those devices are used.

#### Limitations and Future Directions

Due to difficulty of recruiting female digital game players in both experiments the number of participants was unequal, and specifically the number of females was not as high as we hoped. The lack of statistical power is an undeniable limitation of this study, but the convergence in the results gives us confidence in them. However, as the participants were self-selected volunteers, it is possible that, for example, the female sample reflects the general population poorer than the male sample, given that playing digital games is more common in males [31].

We used digital games to set up competitive and cooperative situations and assumed that they constitute an intrinsically motivated activity for participants who (according to their own statement) like to play digital games. Further studies should take into account the explicitly stated motivation (cf. [22]). It has been also shown that competitive orientation affects the intrinsic motivation and the emotional response to competition [8], [19]. For example, it is conceivable that the participants with a high desire to win [19] would have had more positive responses to a more competitive game and to actually winning, whereas the participants with low desire to win could have less positive responses to a more competitive game and perhaps less responsivity in general to winning or losing. Future studies should include a competitiveness measure to control this.

It is possible that the comparisons of EMG activity levels are affected by mostly social smiles instead of smiles resulting from emotions [89]. However, our results indicated that almost actually always males almost always actually smiled more (see Figures 1 and 3), and as female smiles are especially prominent for social situations [42], this speaks against the behavioral ecology interpretation, as that should have led to more smiles in females.

A separate potential limitation related to the tasks and competition rules is the fact that when manipulating cooperative and competitive game conditions, we necessarily also vary the opposition: i.e., as the conflict structure in the game is 2 vs. 2, in cooperative mode the participants are in the same two-character team against two computer-controlled characters, and in competitive mode they are in the opposing teams (with a computer-controlled character). Because of the interconnected structure of the 2 vs. 2 set-up, a purely symmetrical cooperation-competition (where the teammates and the opposition would not also change) was not possible. According to Ravaja and others [86], the (controller of the) opponent has effect on the emotional experience at least in competitive game play. In their study, the human opponent elicited more positive emotions than computer-controlled opponent, and a friend as an opponent elicited also more positive emotions than a stranger and this might have affected our main effects (difference between cooperation and competition) as well. The fact that many emotion and social presence self-reports showed clearly lower ratings when the computer-controlled characters were present (compared to otherwise identical competitive condition where they were not) might suggest that the interconnectedness has had an effect—while on the other hand, the lack of difference in physiological responses implies that this has not affected the general emotional states. With other types of tasks where the interconnectedness between mode and opponent is not an issue (see [22]), there would be no need for computer-controlled team members—although this would also severely limit the types of competition.

It has been shown that personal relationship and mediation may affect emotional reactions to digital games [64], [85], [86], [90], [91]. As in our experiment the participants were friends and they sat side by side in the same room, it is possible that the emotional responses were more positive due to the closeness of the participants, perhaps in interaction with the cooperation/competition manipulation. In future studies it could be tested if there is interaction between mediation, relationship, and competitiveness, although it should be noted that the relationship also may affect the social reasons to smile [42].

#### Conclusions

Our study is the first to examine the gender differences in emotional responses to cooperation and competition. We found that males experience the competition more positively than cooperation, that females do not have different emotional reactions to competition and cooperation, and that the males' probably experienced the competition as more positive than the females experienced either mode. These results suggest that while we consider the activity in our study, playing digital games, intrinsically motivated, the males have higher enjoyment than females, which might lead to the higher preference to competition that has been established in the research literature. The results also do not support the view that females are more cooperative than males, even if they are less competitive, implying that—contrary how they are sometimes discussed—cooperation and competition are not polar opposites.

## Supporting Information

Appendix S1 **Results for body and console movement and social presence evaluations for** **Experiment 1** **, social presence evaluations for** **Experiment 2** **, and post-hoc analyses for potential explanators for gender differences in both experiments.** Also data availability statement.
(DOCX)

Appendix S2 **In accordance with the guidelines of PLOS ONE concerning data availability, Appendix S2 contains the final SPSS files for Experiments 1 and 2.** The raw data can be made available by request.
(ZIP)
