# Supplementary material for: Gender Differences in Emotional Responses to Cooperative and Competitive Game Play
Source: PLoS One. 2014 Jul 1;9(7):e100318. doi: 10.1371/journal.pone.0100318 (PMC4077576; doi:10.1371/journal.pone.0100318)
Supplement: Appendix S1 — Results for body and console movement and social presence evaluations for Experiment 1 , social presence evaluations for Experiment 2 , and post-hoc analyses for potential explanators for gender differences in both experiments. Also data availability statement. (DOCX) [file pone.0100318.s001.docx]

# **Experiment 1**

**Other findings.** Interestingly, both body and console movement (Table S1) showed a clear interaction between gender and mode, with males showing higher levels of body (*p* < .001, Figure S1, left panel) and console (*p* = .001, Figure S1, right panel) movements during competition than cooperation, whereas no difference was found for females. The interaction may reflect increased arousal, as participants might have moved more when more excited, increased positive emotions, according to the activation pattern closely resembling those of Hypothesis 1, or both.

| Table S1. | |  |  |  |  |  |
| --- | --- | --- | --- | --- | --- | --- |
| *Experiment 1 Linear Mixed Models for Physiological Dependent Variables* | | | | | | |
|  |  | Estimated Marginal Means (*SE*) | |  |  |  |
| Model Variables | | 1 | 2 | *df* | *F* | *p* |
| Body movement (ln[g]) | | |  |  |  |  |
|  | Mode | 0.619 (0.008) | 0.639 (0.008) | 1,130.604 | 12.317 | 0.001 |
|  | Gender | 0.647 (0.009) | 0.611 (0.011) | 1,54.705 | 6.092 | 0.017 |
|  | Mode × Gender | 0.626 (0.009) | 0.667 (0.009) | 1,130.604 | 15.913 | **<.001** |
|  |  | 0.613 (0.012) | 0.610 (0.012) | |  |  |
| Console movement (ln[g]) | | |  |  |  |  |
|  | Mode | 0.835 (0.017) | 0.851 (0.017) | 1,122.4 | 2.734 | 0.101 |
|  | Gender | 0.898 (0.021) | 0.788 (0.026) | 1,46.677 | 10.799 | 0.002 |
|  | Mode × Gender | 0.873 (0.022) | 0.923 (0.022) | 1,122.4 | 11.505 | 0.001 |
|  |  | 0.797 (0.027) | 0.779 (0.027) | |  |  |
| *Note*. Intercept is left out as uninformative. Means 1 and 2 correspond to Cooperative and Competitive for Mode, and Male and Female for Gender, respectively. For Mode × Gender interactions, the rows denote Male and Female, and the columns denote Cooperative and Competitive, in that order. False discovery rate is controlled with the same FDR calculation as in main paper. | | | | | | |

Social Presence evaluations (Table S2) demonstrated that especially females, but also males, reported less Empathy after competitive than cooperative game modes (*p* < .001)—possibly because in competition, participants feel having opposite feelings due to generally trying to achieve opposite goals (with items like “When I was happy, the other was happy”). In addition, while the females seem to be more connected with each other’s actions during cooperation, the reverse was true for males (*p* < .001 for Behavioral Involvement), perhaps further reflecting the male preference for competition.

Apparently contradicting the lack of difference in negative emotions between modes (RQ1), males reported more SPGQ Negative Feelings towards the other player after competitive than cooperative mode (*p* = .001). However, it seems possible that this disconnect is explained by a possibility that these typical competitive feelings (e.g., “I felt jealous about the other”, “I felt revengeful” and “I felt schadenfreude”; cf. Vorderer et al., 2003) are not experienced negatively, but actually contribute to the overall positive experience in the intrinsically motivated playful context (see Bartsch et al., 2008, for the concept of meta-emotions during media experiences).

| Table S2. | |  |  |  |  |  |
| --- | --- | --- | --- | --- | --- | --- |
| *Experiment 1 Linear Mixed Models for Self-Report Dependent Variables* | | | | | | |
|  |  | Estimated Marginal Means (*SE*) | |  |  |  |
| Model Variables | | 1 | 2 | *df* | *F* | *p* |
| SPGQ Empathy | |  |  |  |  |  |
|  | Mode | 3.335 (0.084) | 2.827 (0.083) | 1,131.824 | 49.855 | <.**001** |
|  | Gender | 3.034 (0.092) | 3.128 (0.119) | 1,58.811 | 0.39 | 0.535 |
|  | Mode × Gender | 3.151 (0.102) | 2.917 (0.101) | 1,131.824 | 14.507 | <.**001** |
|  |  | 3.519 (0.133) | 2.737 (0.131) | |  |  |
| SPGQ Negative Feelings | | |  |  |  |  |
|  | Mode | 2.263 (0.076) | 2.672 (0.075) | 1,129.128 | 26.685 | <**.001** |
|  | Gender | 2.657 (0.079) | 2.278 (0.101) | 1,57.763 | 8.729 | 0.005 |
|  | Mode × Gender | 2.315 (0.093) | 2.999 (0.092) | 1,129.128 | 12.064 | 0.001 |
|  |  | 2.211 (0.120) | 2.345 (0.118) | |  |  |
| SPGQ Behavioral Involvement | | |  |  |  |  |
|  | Mode | 2.971 (0.094) | 2.904 (0.092) | 1,127.035 | 0.669 | 0.415 |
|  | Gender | 3.267 (0.102) | 2.608 (0.132) | 1,54.61 | 15.654 | <.**001** |
|  | Mode × Gender | 3.080 (0.114) | 3.454 (0.113) | 1,127.035 | 28.801 | <.**001** |
|  |  | 2.862 (0.148) | 2.353 (0.146) | |  |  |
| *Note*. Intercept is left out as uninformative. Means 1 and 2 correspond to Cooperative and Competitive for Mode, and Male and Female for Gender, respectively. For Mode × Gender interactions, the rows denote Male and Female, and the columns denote Cooperative and Competitive, in that order. False discovery rate is controlled with the same FDR calculation as in main paper. | | | | | | |

**Covariate models.** As the later experimental periods elicited higher positive affect compared to earlier periods, and that home was experienced more positively than the laboratory, the models also showed the additional effects for the Social Presence questionnaires for the order of mode, all showing higher values in later periods, ps = .034 (n.s.), .001, and .006, for Empathy, Negative Feelings, and Behavioral Involvement, respectively. It seems plausible that the later experimental periods and periods at home have felt more comfortable and relaxed, allowing higher positive affect.

# **Experiment 2**

Similarly as in Experiment 1, SPGQ Negative Feelings scale followed the physiological measures of positive emotions closely (Table S3): males gave higher ratings in competitive conditions, but the same did not hold true for females (*p* = .003). This result did not remain when comparing to the covariate model, however (*p* > .2, n.s.), while the main effect for condition did (higher ratings in competitive conditions, *p* = .009). Game result was only weakly associated with this subscale, (*F*(2,221.489) = 3.416, *p* = .035), but as the only covariate even close to significance may have affected the SPGQ ratings.

SPGQ Empathy and Behavioral Involvement and Perceived Comprehension were the highest in cooperation and lowest in competition, but there were no significant gender effects.

| Table S3. | |  |  |  |  |  |  |  |
| --- | --- | --- | --- | --- | --- | --- | --- | --- |
| *Linear Mixed Models for Self-Report Dependent Variables in Experiment 2* | | | | | | |  |  |
|  |  | Estimated Marginal Means (*SE*) | | |  |  |  |  |
| Model Variables | | 1 | 2 | 3 | 4 | *df* | *F* | *p* |
| SPGQ Empathy | |  |  |  |  |  |  |  |
|  | Condition | 3.936 (0.067) | 3.777 (0.072) | 3.383 (0.067) | 3.301 (0.067) | 3,222.649 | 38.735 | **<.001** |
|  | Gender | 3.491 (0.067) | 3.707 (0.082) | |  | 1,79.627 | 4.161 | 0.045 |
|  | Condition × Gender | 3.893 (0.085) | 3.633 (0.094) | 3.219 (0.085) | 3.221 (0.085) | 3,222.649 | 1.306 | 0.273 |
|  |  | 3.979 (0.104) | 3.922 (0.11) | 3.547 (0.104) | 3.38 (0.104) |  |  |  |
| SPGQ Negative Feelings | | |  |  |  |  |  |  |
|  | Condition | 2.86 (0.07) | 2.91 (0.075) | 2.908 (0.07) | 3.083 (0.07) | 3,222.229 | 4.574 | **0.004** |
|  | Gender | 2.845 (0.074) | 3.035 (0.09) |  |  | 1,79.77 | 2.624 | 0.109 |
|  | Condition × Gender | 2.682 (0.089) | 2.718 (0.096) | 2.892 (0.089) | 3.09 (0.089) | 3,222.229 | 4.918 | **0.003** |
|  |  | 3.038 (0.109) | 3.101 (0.114) | 2.925 (0.109) | 3.075 (0.109) | |  |  |
| SPGQ Behavioral Involvement | | |  |  |  |  |  |  |
|  | Condition | 4.11 (0.082) | 3.815 (0.088) | 3.435 (0.081) | 3.858 (0.082) | 3,222.524 | 19.997 | **<.001** |
|  | Gender | 3.921 (0.078) | 3.688 (0.095) | |  | 1,79.05 | 3.604 | 0.061 |
|  | Condition × Gender | 4.163 (0.104) | 3.952 (0.114) | 3.469 (0.103) | 4.101 (0.104) | 3,222.524 | 2.332 | 0.075 |
|  |  | 4.057 (0.126) | 3.678 (0.134) | 3.401 (0.126) | 3.615 (0.126) | |  |  |
| Perceived Comprehension | | |  |  |  |  |  |  |
|  | Condition | 4.17 (0.074) | 4.077 (0.079) | 3.66 (0.074) | 3.738 (0.074) | 3,221.554 | 21.823 | **<.001** |
|  | Gender | 3.927 (0.074) | 3.895 (0.09) |  |  | 1,78.607 | 0.074 | 0.787 |
|  | Condition × Gender | 4.183 (0.094) | 4.01 (0.102) | 3.673 (0.093) | 3.842 (0.094) | 3,221.554 | 1.556 | 0.201 |
|  |  | 4.156 (0.114) | 4.144 (0.121) | 3.647 (0.114) | 3.634 (0.114) | |  |  |
| *Note*. Intercept is left out as uninformative from. Means 1 through 4 correspond to Cooperative, Cooperative and Competitive, Competitive, and Competitive Without Computer. Means 1 and 2 correspond to Male and Female for Gender. For Gender × Condition interactions, the rows denote Male and Female and the columns denote the Condition. Note that the figures here are from the basic models without the confounding game result as covariate. False discovery rate is controlled with the same FDR calculation as in main paper. | | | | | | | | |

# **Potential explanators for gender differences: Experiments 1 & 2**

As can be seen from Table S4, while in Experiment 1 the Anticipated Threat ratings were very similar across genders and modes, Stress was anticipated to be higher in competition (compared to cooperation, *p* < .001). The general level of Anticipated Stress also seemed higher in females, but neither that nor the interaction were significant (*p*s > .1). In Anticipated Success there was a slight difference in gender main effect, males showing higher ratings (indicating higher self-confidence, *p* = .048) than females, but when we re-ran the analysis with previous experience with game as a covariate, the effect disappeared (*p* = .211). BIS sensitivity was not significant either (difference in estimates 0.18 points on scale from 1 to 5, *F*(1,44) = 0.974, *p* = .329).

The same analyses were run for Experiment 2 data (Table S5). Anticipated Threat was highest in competition and lowest in cooperation (*p* = .012), but the gender difference was not significant (*p* = .092 for main effect, *p* = .117 for interaction). Anticipated Stress showed the same pattern but stronger: highest in competition and lowest in cooperation (*p* = .005) and higher for males than females (*p* = .011, interaction ns.), and while controlling the previous experience weakened the gender effect, it remained significant (Ms 1.682 (0.138 *SE*) for males and 2.261 (0.177 *SE*) for females, *F*(79.835) = 5.343, *p* = .023). BIS sensitivity was also higher than in Experiment 1, the difference in estimates being 0.34 higher in females, *F*(1,80) = 12.475, *p* = .001, but there were no gender difference in BAS sensitivity (*p* > .05). Due to these found differences, BIS sensitivity and anticipated stress were tested as covariates for Hypothesis 1 tests for ZM EMG, but that did not affect the main results (*p* < .001 for the mode × gender interactions).

Anticipated Success showed a negligible linear decrease from Condition 1 through 4 (ns), and higher ratings for males than females (*p* = .013, interaction ns.), but this effect was also lost when the previous experience was controlled (Ms = 3.532 for male, 3.558 for female, *F*(1,80.074) = 0.017, *p* = .897).

We interpret that females might be higher in Stress (estimated difference about half a point on a 5-point scale for main effect – note that this did not hold for interaction), manifesting their higher BIS sensitivity (the gender difference in Anticipated Stress was detected only when there was also a difference in BIS sensitivity). There probably was not a difference in threat appraisal or self-confidence, as the gender differences in the latter were explained by the gender differences in previous experience of the game.

| Table S4. |  |  |  |  |  |  |
| --- | --- | --- | --- | --- | --- | --- |
| *Experiment 1 Post-Hoc Linear Mixed Models for Self-Report Dependent Variables* | | | | | | |
|  |  | Estimated Marginal Means (*SE*) | |  |  |  |
| Model Variables | | 1 | 2 | *df* | *F* | *p* |
| Anticipated Threat | |  |  |  |  |  |
|  | Mode | 1.400 (0.108) | 1.583 (0.108) | 1,143.287 | 3.849 | 0.052 |
|  | Gender | 1.508 (0.119) | 1.475 (0.154) | 1,48.158 | 0.029 | 0.865 |
|  | Mode × Gender | 1.383 (0.132) | 1.633 (0.132) | 1,143.287 | 0.507 | 0.478 |
|  |  | 1.417 (0.171) | 1.534 (0.172) | |  |  |
| Anticipated Stress | |  |  |  |  |  |
|  | Mode | 2.017 (0.154) | 2.582 (0.154) | 1,143.345 | 16.896 | <.001 |
|  | Gender | 2.092 (0.169) | 2.507 (0.218) | 1,48.206 | 2.266 | 0.139 |
|  | Mode × Gender | 1.783 (0.188) | 2.400 (0.188) | 1,143.345 | 0.142 | 0.707 |
|  |  | 2.250 (0.243) | 2.763 (0.245) | |  |  |
| Anticipated Success | |  |  |  |  |  |
|  | Mode | 4.275 (0.172) | 4.204 (0.173) | 1,140.049 | 0.289 | 0.592 |
|  | Gender | 4.562 (0.195) | 3.917 (0.252) | 1,47.981 | 4.102 | 0.048 |
|  | Mode × Gender | 4.517 (0.210) | 4.607 (0.210) | 1,140.049 | 1.51 | 0.221 |
|  |  | 4.032 (0.272) | 3.801 (0.274) | |  |  |
| *Note*. Intercept is left out as uninformative. Means 1 and 2 correspond to Cooperative and Competitive for Mode, and Male and Female for Gender, respectively. For Mode × Gender interactions, the rows denote Male and Female, and the columns denote Cooperative and Competitive, in that order. The false discovery rate of post-hoc tests is not controlled. | | | | | | |

| Table S5. | |  |  |  |  |  |  |  |
| --- | --- | --- | --- | --- | --- | --- | --- | --- |
| *Experiment 2 Post-Hoc Linear Mixed Models for Self-Report Dependent Variables* | | | | | | |  |  |
|  |  | Estimated Marginal Means (*SE*) | | |  |  |  |  |
| Model Variables | | 1 | 2 | 3 | 4 | *df* | *F* | *p* |
| Anticipated Threat | |  |  |  |  |  |  |  |
|  | Condition | 1.646 (0.119) | 1.839 (0.119) | 2.014 (0.119) | 1.927 (0.119) | 3,234.991 | 3.75 | 0.012 |
|  | Gender | 1.692 (0.122) | 2.020 (0.149) | |  | 1,79.450 | 2.904 | 0.092 |
|  | Condition × Gender | 1.479 (0.153) | 1.646 (0.150) | 1.729 (0.150) | 1.916 (0.151) | 3,234.991 | 1.984 | 0.117 |
|  |  | 1.812 (0.184) | 2.031 (0.184) | 2.299 (0.185) | 1.937 (0.184) | |  |  |
| Anticipated Stress | |  |  |  |  |  |  |  |
|  | Condition | 1.770 (0.116) | 1.911 (0.115) | 2.096 (0.116) | 2.082 (0.116) | 3,235.241 | 4.369 | 0.005 |
|  | Gender | 1.712 (0.122) | 2.217 (0.150) | |  | 1,79.794 | 6.835 | 0.011 |
|  | Condition × Gender | 1.571 (0.148) | 1.667 (0.146) | 1.729 (0.146) | 1.882 (0.147) | 3,235.241 | 1.146 | 0.331 |
|  |  | 1.969 (0.179) | 2.156 (0.179) | 2.463 (0.180) | 2.281 (0.179) | |  |  |
| Anticipated Success | |  |  |  |  |  |  |  |
|  | Condition | 3.641 (0.108) | 3.505 (0.107) | 3.452 (0.107) | 3.416 (0.107) | 3,235.675 | 1.564 | 0.199 |
|  | Gender | 3.713 (0.105) | 3.294 (0.128) | |  | 1,80.002 | 6.407 | 0.013 |
|  | Condition × Gender | 3.969 (0.138) | 3.729 (0.135) | 3.604 (0.135) | 3.551 (0.136) | 3,235.675 | 1.24 | 0.296 |
|  |  | 3.313 (0.165) | 3.281 (0.165) | 3.299 (0.167) | 3.281 (0.165) | |  |  |
| *Note*. Intercept is left out as uninformative from all models. Means 1 through 4 correspond to Cooperative, Cooperative and Competitive, Competitive, and Competitive Without Computer. Means 1 and 2 correspond to Male and Female for Gender. For Gender × Condition interactions, the rows denote Male and Female and the columns denote the Condition. Note that the figures here are from the basic models without the confounding game result as covariate. The false discovery rate of post-hoc tests is not controlled. | | | | | | | | |
